# DM-Net: a physics-model-independent direct mapping approach for calibration-free multi-coil MRI

**DOI:** 10.21203/rs.3.rs-7174070/v1

**Published:** 2025-09-01

**Authors:** Yan Wu, Cagan Alkan, Julio Oscanoa, Aiqi Sun, Kawin Setsompop, Ali Syed, Yajun Ma, Congyu Liao, Marcus Alley, Fan Zhang, John Pauly, Shreyas Vasanawala

**Affiliations:** 1 Stanford University; 2 University of California, San Diego

## Abstract

Deep learning-based multi-coil magnetic resonance image reconstruction has been actively investigated and increasingly applied in clinical settings. However, most models are physics-model-based approaches, which either require precalculation of coil sensitivity or fail to achieve optimal performance without taking coil sensitivity into consideration.

Inspired by ESPIRiT, we propose a physics-model-independent direct mapping approach, namely DM-Net, which aims for optimal reconstruction without explicitly using coil sensitivity. A densely connected convolutional network is employed, where coil sensitivity and channel correlation are intrinsically exploited.

For comparison, other reconstruction models that mimic SENSE and GRAPPA are implemented. DMwS-Net is a direct mapping approach that incorporates precalculated coil sensitivity maps as additional input; and CCR-Nets provide coil-by-coil reconstruction, where individual coil images are jointly predicted and combined with or without coil sensitivity.

The proposed models are trained and tested on 5440 images from 17 subjects acquired with 3DFT (Fourier transform). DM-Net achieves superior performance, suggesting the feasibility of excluding precalculated coil sensitivity from model input. Moreover, DM-Net can be applied on k-space data without a fully sampled calibration region, which may improve image quality by sampling high frequency regions more densely.

This work expands the scope of DL-based image reconstruction and provides evidence that a direct mapping framework without using coil sensitivity may lead to reliable, simplified and faster reconstruction.

## Introduction

Magnetic Resonance Imaging (MRI) is a powerful but relatively slow imaging modality. To accelerate data acquisition, undersampling in k-space is typically applied, which requires data interpolation in image reconstruction. In a multi-coil MRI system, channel correlation and coil sensitivity are valuable information that can be used to exploit data redundancy in the coil dimension, where parallel imaging methods were developed [1–3].

**Figure 1.**
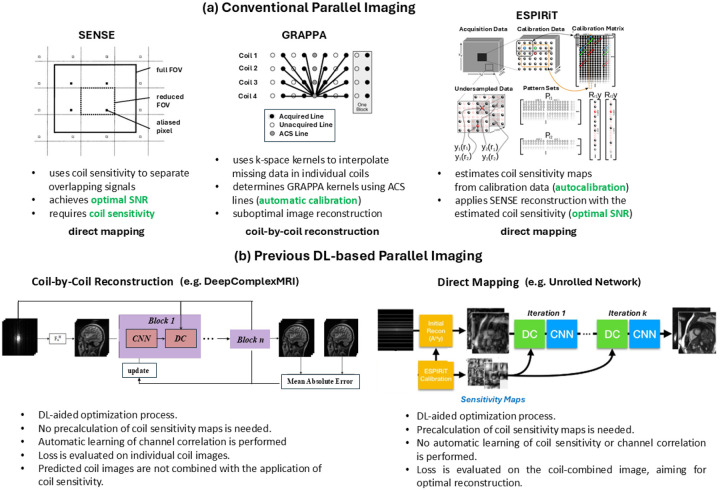
Previous conventional and DL-based parallel imaging techniques. **(a)** Conventional parallel imaging techniques: SENSE, GRAPPA and ESPIRiT. **(b)** Previous DL-based parallel imaging techniques: coil-by-coil reconstruction (e.g. DeepComplexMRI) and physics-model-based direct mapping (e.g. unrolled network).

Conventional parallel imaging methods can be categorized into coil-by-coil reconstruction and direct mapping approaches, depending on whether individual coil images are reconstructed along with the coil-combined image. Common methods include SENSE, GRAPPA, and ESPIRiT, which work either in the image domain or in k-space (Fig. 1). In particular, SENSE reconstructs a coil-combined image by using coil sensitivity to separate overlapping signals in the image domain [1]. It achieves optimal SNR but requires *a priori* knowledge on coil sensitivity, which can be acquired via a separate scan but may be misaligned with multi-coil data if there is motion. Alternatively, GRAPPA jointly reconstructs individual coil images by using k-space kernels to interpolate missing data points in every individual coil and then combines the reconstructed coil images with root-of-sum-of-squares [2]. While GRAPPA kernels are automatically calibrated, the coil-combined image obtained without using coil sensitivity does not have optimal SNR. Taking advantages of both methods, ESPIRiT estimates coil sensitivity maps from fully sampled center k-space calibration data and reconstructs a coil-combined image in the way that SENSE does, thus achieving automatic calibration and optimal SNR [3].

More recently, deep learning-based multi-coil image reconstruction methods have been developed, which can also be categorized into coil-by-coil reconstruction and direct mapping models [4, 5].

RAKI and DeepComplexMRI are examples of coil-by-coil reconstruction models [6, 7]. RAKI is a scan specific model that is trained on calibration data [6]. Instead of manually calculating convolutional kernels, a convolutional neural network is automatically learned to produce coil images where the loss is evaluated between the reconstructed and acquired k-space lines in the calibration region. Conversely, DeepComplexMRI reconstructs individual coil images in a DL-aided optimization framework, where an objective function is minimized by enforcing data consistency and using deep network prediction as a regularization term [7]. While channel correlation is automatically learned, DeepComplexMRI does not provide optimal coil-combined image reconstruction without using coil sensitivity.

Unrolled networks are representatives of direct mapping approaches [8, 9]. Without reconstructing individual coil images, these models directly estimate a coil-combined image in a DL-aided optimization framework with physics models embedded. Here, coil sensitivity maps are required – they are applied on the estimated coil-combined image such that individual coil images are generated for data consistency enforcement in the next iteration. Due to the use of a coil-combined image as the input, these deep learning models cannot automatically learn channel correlation or coil sensitivity maps (even though coil sensitivity maps can be explicitly estimated using a separate model, such as deepSENSE [10]).

In previous deep learning-based image reconstruction models, coil sensitivity and channel correlation are not automatically exploited. Physics-model-based direct mapping approaches typically require precalculated coil sensitivity maps, whereas coil-by-coil reconstruction models do not take coil sensitivity into consideration.

We aim for optimal reconstruction without explicitly using coil sensitivity maps. A deep learning-based physics-model-independent direct mapping approach, namely DM-Net, is proposed, which employs a densely connected convolutional network to provide an end-to-end mapping from undersampled multi-coil images to a high-quality coil-combined image. DM-Net is a pure deep learning framework, where deep learning plays a broader role than just acting as a regularization term in a DL-aided optimization method. We hypothesize that coil sensitivity maps and channel correlations may be implicitly estimated within the DM-Net.

To assess comparative performance, other physics-model-independent deep learning methods are developed. We investigate whether incorporation of precalculated coil sensitivity maps into a direct mapping framework improves the prediction accuracy. Moreover, coil-by-coil reconstruction models are constructed, where individual coil images are predicted and combined with or without using coil sensitivity. Here, the application of coil sensitivity is motivated by a promise of improving SNR and avoiding magnitude noise bias in the coil combination process for low SNR data.

## Methods

### Overview

**Figure 2.**
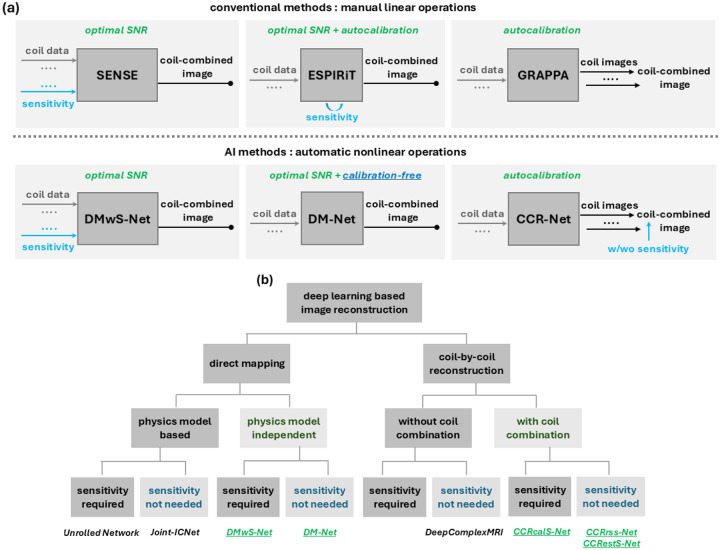
Proposed methods as compared to conventional parallel imaging techniques and previous deep learning models. **(a)** The proposed deep learning methods have similar input and output as conventional parallel imaging approaches. Specifically, DM-Net is parallel to ESPIRiT, which is a direct mapping approach that does not require coil sensitivity maps as model input; DMwS-Net, like SENSE, also performs direct mapping but requires coil sensitivity as input; CCR-Nets are coil-by-coil methods like GRAPPA, which may or may not use precalculated coil sensitivity maps for coil combination. **(b)** The proposed methods expand the scope of deep learning-based image reconstruction techniques to new areas. Particularly, DM-Net and DMwS-Net are physics-model-independent direct mapping, whereas variants of CCR-Nets (i.e., CCRrss-Net, CCRcalS-Net, and CCRestS-Net) are coil-by-coil reconstruction models that perform coil combination in different ways.

In this study, we propose a deep learning-based physics-model-independent direct mapping approach, DM-Net, for calibration-free MR imaging. DM-Net performs optimal reconstruction without requiring coil sensitivity maps as model input. In this sense, it resembles ESPIRiT (Fig. 2a).

In addition, other physics-model-independent deep learning approaches that are parallel to SENSE or GRAPPA are developed. In particular, DMwS-Net is a direct mapping model with coil sensitivity maps incorporated as additional model input, and CCR-Nets are coil-by-coil reconstruction models where individual coil images are jointly predicted and combined such that loss can be evaluated on the coil-combined image as well.

In fact, these models expand the scope of deep learning-based image reconstruction techniques to less investigated areas (Fig. 2b). While previous direct mapping models were physics-model-based, DM-Net and DMwS-Net do not rely on physics models (with or without precalculated coil sensitivity maps as model input). In addition, the proposed coil-by-coil reconstruction models perform coil combination (CCRrss-Net conducts root-of-sum-of-squares, whereas CCRcalS-Net and CCRestS-Net use precalculated or estimated coil sensitivity maps).

### DL-based Direct Mapping (DM)

We propose a direct mapping model, DM-Net, which predicts a coil-combined image from undersampled coil images without explicitly using precalculated coil sensitivity maps.

DM-Net can be regarded as parallel to ESPIRiT. As is widely known, a major advantage of ESPIRiT is obviating the measurement of coil sensitivity maps by extracting the information from center k-space calibration data. Similarly, DM-Net does not require precalculation of coil sensitivity maps, which may be implicitly estimated within the model. Moreover, in DM-Net, coil sensitivity is derived using nonlinear operators without a constraint in the dimension of data space (contrarily in ESPIRiT, coil sensitivity is obtained via *linear* eigenvector decomposition in a *subspace* of k-space).

**Figure 3.**
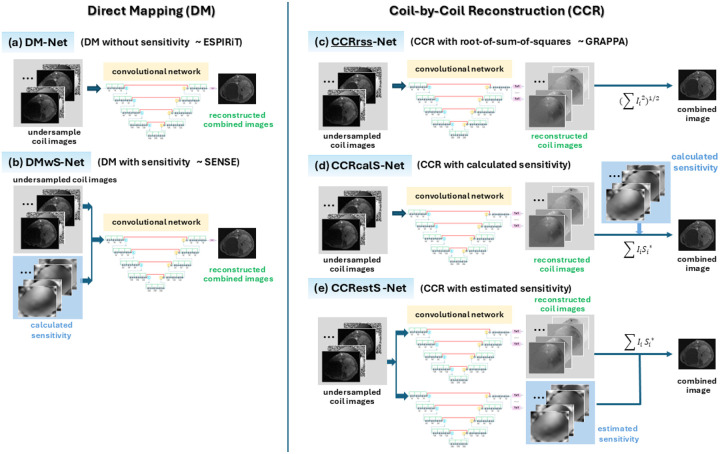
DL-based multi-coil image reconstruction methods. **(a-b)** Direct mapping models which predict a coil-combined image directly from undersampled multi-coil data. Particularly, DM-Net predicts a coil-combined image directly from undersampled multi-coil images; DMwS-Net predicts a coil-combined image from undersampled multi-coil images and precalculated coil sensitivity maps, where the precalculated coil sensitivity maps are implicitly used to form a coil-combined image. **(c-e)** Coil-by-coil reconstruction models where individual coil images are jointly predicted from undersampled k-space data and combined in different ways. In particular, CCRrss-Net combines the reconstructed coil images via root-of-sum-of-squares; CCRcalS-Net applies the precalculated coil sensitivity maps in coil combination; whereas CCRestS-Net predicts both coil images and coil sensitivity maps, and combines the reconstructed coil images with the application of estimated coil sensitivity maps.

In DM-Net, a densely connected convolutional network [11] is employed to provide direct mapping from undersampled multi-coil data to a coil-combined image (Fig. 3a). The input is the magnitude and phase images obtained from undersampled k-space data using inverse Fast Fourier Transform. The combination of magnitude/phase images works better than real/imaginary images based on our experience. Coil sensitivity maps are not taken as the input, but hypothesized to be implicitly estimated and automatically applied, since they are presumably needed for optimal reconstruction.

Additionally, DMwS-Net is developed as a direct mapping model that reconstructs a coil-combined image from both coil sensitivity maps and multi-coil data (Fig. 3b). Incorporation of precalculated coil sensitivity maps as additional input is a flexible way of providing *a priori* information, which will be implicitly used to form a coil-combined image with possible improvement in prediction accuracy. But if similar performance is achieved, one can infer that precalculation of coil sensitivity maps is no longer needed.

### DL-based Coil-by-Coil Reconstruction (CCR)

For comparison purposes, coil-by-coil reconstruction models CCR-Nets are constructed, where individual coil images are jointly reconstructed and combined.

CCR-Nets are parallel to GRAPPA, but have significantly larger numbers of convolution operators, which determine the degree of freedom when approximating the reconstruction function. For this reason, CCR-Nets are expected to improve representation of the reconstruction function than GRAPPA and RAKI.

In CCR-Nets, a multi-output densely connected convolutional network is employed to provide mapping from undersampled multi-coil data to high-quality individual coil images. Here, the magnitude and phase images obtained with inverse Fast Fourier Transform are taken as the input, whereas the real/imaginary images are predicted as the output (so as to remove the constraint on the predicted values, i.e., -π to π for phase, non-negative for magnitude). During the joint reconstruction of individual coil images, channel correlation is automatically exploited.

Then the reconstructed coil images are combined in CCR-Nets. In this way, the loss can be evaluated on the coil-combined image (ultimate goal) as well as on individual coil images. This is different from previous coil-by-coil reconstruction models, where loss is only evaluated on individual coil images [7].

We start with CCRrss-Net (CCR with root-of-sum-of-squares), which performs root-of-sum-of-squares to form a coil-combined image (Fig. 3c). Since CCRrss-Net does not provide optimal reconstruction without using coil sensitivity maps, the model is further modified to apply coil sensitivity maps in coil combination (as motivated by a promise of improving SNR and avoiding magnitude noise bias in the coil combination process for low SNR data). One way is CCRcalS-Net (CCR with precalculated sensitivity), where precalculated coil sensitivity maps are used to combine the reconstructed coil images (Fig. 3d). Another option is CCRestS-Net (CCR with estimated sensitivity), where coil sensitivity maps are simultaneously estimated and explicitly applied on the reconstructed coil images (Fig. 3e).

### MIMO/MISO Reconstructions and Loss Functions

Multi-coil images are taken as the input in the proposed direct mapping and coil-by-coil reconstruction models. It is important not to merge multi-coil data into a coil-combined image as the network input. Otherwise, the deep neural network cannot automatically exploit channel correlation and coil sensitivity from a single coil-combined input image.

Different models produce different outputs. The direct mapping models are multi-input single-output (MISO) systems that predict a single coil-combined image. Conversely, the coil-by-coil reconstruction models are multi-input multi-output (MIMO) systems that derive multiple coil images.

In the direct mapping models, the loss is the measured as the difference between the predicted and ground truth coil-combined image: $l_{1,\text{DM}}=\left|\overset{ˆ}{\text{y}}\left(x_{1}\ldots x_{n},\theta \right)-y\right|$ and $l_{1,\text{DMwS}}=\left|\overset{ˆ}{\text{y}}\left(x_{1}\ldots x_{n},s_{1}\ldots s_{n},\theta \right)-y\right|$. For an individual coil $i,x_{i}$ is the undersampled coil image, $s_{i}$ is the coil sensitivity map precalculated using ESPIRiT, θ is the network parameters, $\hat{\text{y}}\left(x_{1}\ldots x_{n},\theta \right)$ is the predicted coil-combined image, and y is the ground truth coil-combine image obtained from fully sampled k-space data.

In the coil-by-coil reconstruction models, the loss is evaluated on individual coil images, coil-combined image, and estimated coil sensitivity maps if applicable. For an individual coil $i,y_{\text{i}}$ and ${\hat{\text{y}}}_{\text{i}}\left(x_{1}\ldots x_{n},\theta \right)$ are the ground truth and predicted individual coil images, $s_{\text{i}}$ and ${\hat{\text{s}}}_{\text{i}}\left(x_{1}\ldots x_{n},\theta \right)$ are the precalculated and predicted coil sensitivity maps.


$$l_{1,\text{CCRrss}}=\sum_{i=1}^{n}\left|{\overset{ˆ}{\text{y}}}_{\text{i}}\left(x_{1}\ldots x_{n},\theta \right)-y_{\text{i}}\right|+{\left(\sum_{i=1}^{n}{\left|{\overset{ˆ}{\text{y}}}_{\text{i}}\left(x_{1}\ldots x_{n},\theta \right)\right|}^{2}\right)}^{1/2}-{\left(\sum_{i=1}^{n}{\left|y_{\text{i}}\right|}^{2}\right)}^{1/2}.$$

$$l_{1,\text{CCRcalS}}=\sum_{i=1}^{n}\left|{\hat{\text{y}}}_{\text{i}}\left(x_{1}\ldots x_{n},\theta \right)-y_{\text{i}}\right|+\left|\sum_{i=1}^{n}{\hat{\text{y}}}_{\text{i}}\left(x_{1}\ldots x_{n},\theta \right)\cdot {s_{i}}^{*}-\sum_{i=1}^{n}y_{\text{i}}\cdot {s_{i}}^{*}\right|.$$

$$l_{1,\text{CCRestS}}=\sum_{i=1}^{n}\left|{\hat{\text{y}}}_{\text{i}}\left(x_{1}\ldots x_{n},\theta \right)-y_{\text{i}}\right|+\left|\sum_{i=1}^{n}{\hat{\text{y}}}_{\text{i}}\left(x_{1}\ldots x_{n},\theta \right)\cdot {\hat{\text{s}}}_{i}{\left(x_{1}\ldots x_{n},\theta \right)}^{*}-\sum_{i=1}^{n}{y_{\text{i}}}^{\cdot }{s_{i}}^{*}\right|+\sum_{i=1}^{n}\left|{\hat{\text{s}}}_{i}\left(x_{1}\ldots x_{n},\theta \right)-s_{i}\right|.$$

### Calibration Region Undersampling

In an undersampled acquisition, a center k-space region is typically fully sampled for calibration or coil sensitivity estimation [2, 3]. However, the calibration data may account for a large proportion of the acquired data, resulting in a paucity of high frequency data. In addition, fully sampled calibration data are not always available, where the SAKE algorithm was proposed for the derivation of coil sensitivity maps [12].

Here we train a DM-Net for image reconstruction from undersampled k-space data acquired without a fully sampled calibration region. It is compared to another model DMwS-Net whose input includes the same individual coil images as well as precalculated coil sensitivity maps obtained from a fully sampled calibration region. If similar results are obtained, that indicates a fully sampled calibration region is not required for implicit estimation of coil sensitivity maps or optimal reconstruction of the coil-combined image. Undersamping in the center k-space calibration region may lead to improved spatial resolution due to more dense sampling in high frequency regions.

### Network Configuration

A densely connected convolutional network (T-Net [11]) is constructed for image reconstruction (Appendix A).

The network for a direct mapping or coil-by-coil reconstruction model has single or multiple output channels. Each output image is produced by a 1×1 convolutional block at the very end, which merge feature maps into a coil-combined image or the real or imaginary part of an individual coil image.

Most models use a single T-Net, whereas CCRestS-Net employs two parallel T-Nets for coil image reconstruction and coil sensitivity estimation; then the predicted coil sensitivity maps are applied on the reconstructed coil images to form the final coil-combined image. Thus, CCRestS-Net has twice as many parameters as the other models.

### Model Training and Testing

Publicly available fully sampled 3D FSE knee datasets from mridata.org (https://archive.ismrm.org/2018/3425.html) are used for training and testing [13]. It contains datasets from 17 subjects, each having 320 slices with a matrix size of 256 × 320, acquired with an 8-channel coil at 3T.

The 3D datasets are retrospectively undersampled using a variable-density Poisson disk [14] to achieve an effective acceleration factor of 6 or 10, thus reducing the scan time from 66 min to 11 or 6.6 min for a 3D dataset. In particular, a fully sampled 24 × 24 center k-space region is used for calibration, where coil sensitivity maps are obtained using ESPIRiT.

Models are trained and tested using 6-fold cross validation. The loss between the predicted and ground truth images is backpropagated and used to update network parameters with the Adam algorithm [15, 16].

## Results

**Figure 4.**
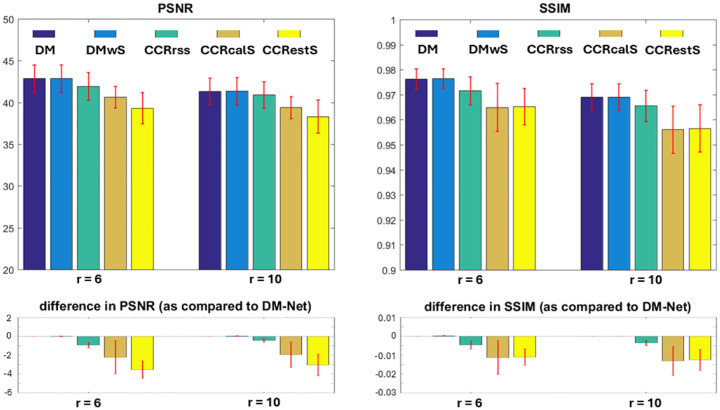
PSNR and SSIM obtained with different DL-based image reconstruction models. The PSNR/SSIM are evaluated per subject with mean and standard deviation shown. Additionally, for every subject, the PSNR/SSIM obtained using each model is compared to the PSNR/SSIM derived using DM-Net. Here the mean values demonstrate the difference between various models, and the error bars show the variation between different subjects. There is statistically significant performance advantage of direct mapping across two accelerations.

**Figure 5.**
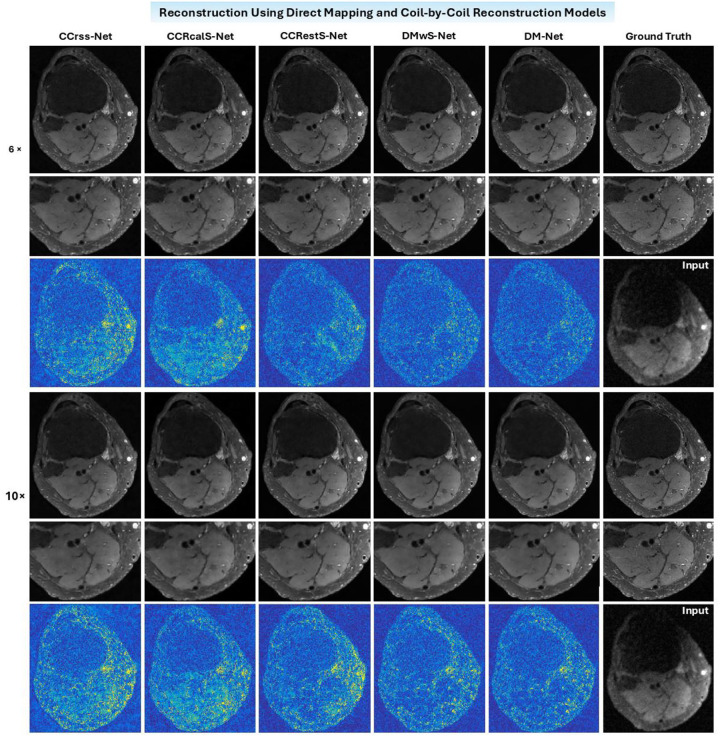
Coil-combined images reconstructed using the proposed direct mapping and coil-by-coil reconstruction models with 6× or 10× acceleration. In each case, different columns demonstrate images derived using coil-by-coil reconstruction models (CCRrss-Net, CCRcalS-Net, CCRestS-Net), direct mapping models (DMwS-Net, DM-Net) as well as the fully sampled ground truth image, and different rows show images, zoomed insets, and amplified error maps (by a factor of 9). Overall, the direct mapping models perform slightly better than coil-by-coil reconstruction models. Even without explicitly using precalculated coil sensitivity maps, DM-Net achieves comparable performance as DMwS-Net.

Using the proposed models, coil-combined images are reconstructed from data with 6× and 10× acceleration. The PSNR and SSIM of resulting magnitude images are shown in Fig. 4, and a representative example is presented in Fig. 5.

Overall, the direct mapping approaches perform better than the coil-by-coil reconstruction models and take less training time due to the complexity of the loss function. In direct mapping, DM-Net achieves comparable performance as DMwS-Net, which indicates that precalculation of coil sensitivity maps can be eliminated. In coil-by-coil reconstruction, the application of coil sensitivity maps does not improve image quality.

**Figure 6.**
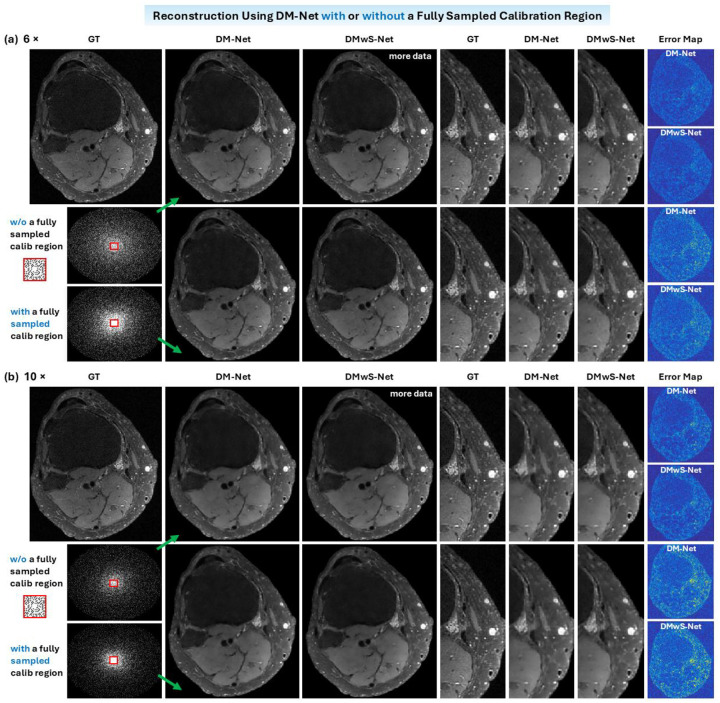
Coil-combined images reconstructed using DM-Net from data acquired with or without a fully sampled calibration region. In the latter case, the sampling rate within the 24 × 24 calibration region is 0.75. The image predicted using DM-Net without a fully sampled calibration region is very close to the one derived using DMwS-Net, even though DMwS-Net takes additional input (precalculated coil sensitivity maps) that contain more center k-space data points. This indicates a fully sampled calibration region is not required for coil-combined image reconstruction or implicit coil sensitivity estimation. At a given acceleration factor, the image derived from data without a fully sampled calibration region (upper row) has improved quality than the one obtained with a fully sampled calibration region (lower row), which can be attributed to more dense sampling in high frequency regions as shown in the sampling patterns (lower left corner).

DM-Nets are also trained to reconstruct coil-combined images from data acquired without a fully sampled calibration region (Fig. 6). Here, the k-sapce is sampled using a variable-density Poisson disk with a 1×1 calibration region, whereas the 24 × 24 center k-space region has a sampling ratio of 0.75. Without a fully sampled calibration region, the coil-combined images predicted using DM-Net are very close to those derived using DMwS-Net, even though DMwS-Net takes additional input (precalculated coil sensitivity maps) which contain more center k-space data points. This indicates a fully sampled calibration region is not required for implicit estimation of coil sensitivity or reconstruction of coil-combined image. At a given acceleration factor, the images derived from data without a fully sampled calibration region have slightly improved image quality than those obtained with a fully sampled calibration region, which can be attributed to more dense sampling in high frequency regions (as shown in the sampling pattern in the lower left corner).

**Figure 7.**
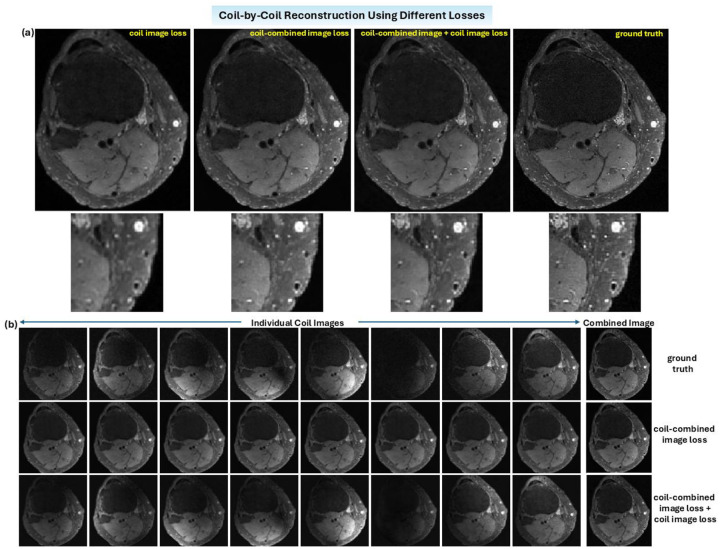
Images predicted using CCRrss-Nets trained with different loss functions. **(a)** Coil-combined images derived using different loss. The highest accuracy is achieved when the loss is evaluated on both coil images and the coil-combined image. **(b)** Individual coil images predicted different losses. If the loss is only evaluated on the coil-combined image, then the reconstructed coil images are identical. This problem can be solved by adding constraints on individual coil images.

**Figure 8.**
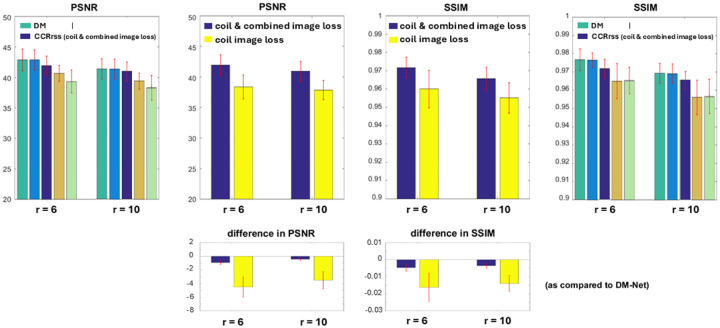
PSNR and SSIM obtained with CCRrss-Nets trained using different loss functions. The PSNR/SSIM per subject are shown, and the difference in PSNR/SSIM between these models and DM-Net are measured. Using CCRrss-Net, the loss evaluated on both coil images and the combined image leads to statistically significant improvements over the conventional loss only evaluated on coil images. However, the upgraded CCRrss-Net is still inferior to the proposed DM-Net.

Additionally, CCRrss-Nets trained with different loss functions are compared (Fig. 7). A higher accuracy is achieved when the loss is evaluated on both the coil-combined image and individual coil images (Fig. 8). The quality of coil-combined image should be assessed since it is the ultimate goal. However, if the loss is only evaluated on the coil-combined image, identical coil images are reconstructed, since the model cannot determine the source of signal. Adding constraints on individual coil images improves the conditioning (Fig. 7b).

## Discussion

Previously, deep learning-based image reconstruction methods were either physics-model-based direct mapping that requires precalculated coil sensitivity maps, or coil-by-coil reconstruction that is suboptimal without taking coil combination into consideration. In this study, we propose physics-model-independent direct mapping and coil-by-coil reconstruction methods that expand the scope of deep learning-based image reconstruction. Different reconstruction strategies implemented on the same deep network architecture are compared. In general, direct mapping performs slightly better than coil-by-coil reconstruction models, because the ground truth coil-combined images have theoretically optimal SNR. Of all the models, DM-Net achieves the best performance with minimal training time.

Even without explicitly using coil sensitivity maps, DM-Net achieves comparable results as DMwS-Net. There are two possible underlying reasons -- coil sensitivity maps are either not needed in optimal reconstruction or implicitly estimated in the reconstruction model. We hypothesize that information on coil sensitivity may be implicitly estimated and automatically utilized, since they are presumably needed for optimal reconstruction. In fact, the capability of deep learning in implicit estimation and integrated use of contributing factors have been demonstrated in previous studies. In quantitative parametric mapping models, magnetic/radiofrequency field maps were implicitly estimated and automatically applied, and thus a *compensated* T1 or T2 map was derived from weighted images without incorporating field maps as the input [17–19]. On the other hand, the provision of *a priori* information as additional model input may improve performance, if the implicit estimation is not accurate [20]. Here, the high performance of DM-Net indicates the accuracy of implicitly estimated coil sensitivity maps.

In DM-Net, coil sensitivity and channel correlation are intrinsically learned. For this reason, the need to precalculate coil sensitivity maps is eliminated, which can simplify the clinical workflow and speed up overall image reconstruction without the expected performance decrease. Simultaneous learning of coil sensitivity and channel correlation have not been fully achieved in earlier studies -- in model-based direct mapping frameworks, neither channel correlation nor coil sensitivity was automatically learned, whereas in previous coil-by-coil reconstruction models, coil sensitivity was not used to combine coil images.

Moreover, we have shown that DM-Net supports reconstruction from k-space data without a fully sampled calibration region. Loosening the constraint on calibration regions is an important advance that may improve the image quality and achievable acceleration factor. The feasibility has been demonstrated by the example that uses a different Poisson disk, but more work is needed to optimize the sampling pattern without such a constraint.

This study has some limitations, and more investigation is required to assess performance with different undersampling patterns, acceleration factors, and pulse sequences. Currently, as the study is restricted to one particular coil array and one body part, generalization needs to be investigated. While quantitative image quality metrics are helpful, radiologist reader studies will also be valuable. In addition, thorough investigation into noise covariance can be carried out.

## Conclusions

We present DM-Net, a physics-model-independent direct mapping approach that does not require precalculated coil sensitivity maps. Achieving performance comparable to other direct mapping and coil-by-coil reconstruction models, this work provides evidence that implicit estimation and integrated use of coil sensitivities in a direct mapping model may lead to reliable MR imaging with simplified and faster reconstruction.

## Figures and Tables

**Figure 9.**
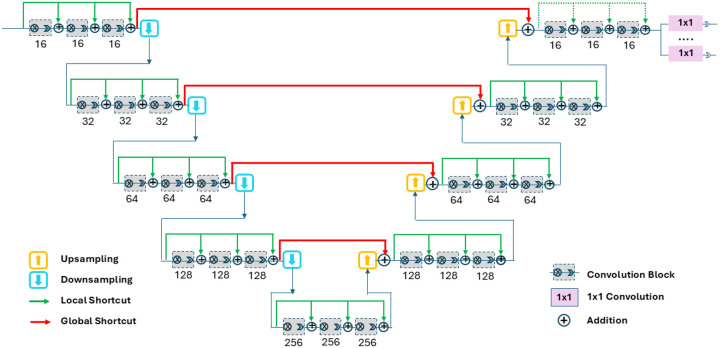
Densely connected convolutional network (T-Net). It was modified from U-Net with densely connected shortcuts added to facilitate more effective residual learning. The network has 1×1 convolutional blocks at the very end, producing a single coil-combined image (with magnitude and phase channels) in a direct mapping model or multiple coil images in a coil-by-coil reconstruction model.

## Appendix

A densely connected convolutional network (T-Net [11]) is constructed for image reconstruction, which was a variation of U-Net and V-Net [21–25] (Fig. 9). The hierarchical network architecture supports feature extraction at different scales, whereas densely connected local shortcut connections facilitate more effective residual learning. With 1×1 convolutional blocks employed at the very end, the T-Net produces single or multiple complex images for direct mapping or coil-by-coil reconstruction.

In fact, the direct mapping and coil-by-coil reconstruction framework can be implemented on any deep neural network that is capable of image-to-image translation. If SAT-Net [26] is adopted, the performance would be further improved due to the use of the self-attention mechanism (which captures long-range dependency) [27]. Similarly, using more sophisticated losses (e.g., a combination of *l*_1_ loss and SSIM [11]) is helpful.
